# Targeting the delivery of dietary plant bioactives to those who would benefit most: from science to practical applications

**DOI:** 10.1007/s00394-019-02075-5

**Published:** 2019-10-22

**Authors:** Baukje de Roos, Anna-Marja Aura, Maria Bronze, Aedin Cassidy, María-Teresa Garcia Conesa, Eileen R. Gibney, Arno Greyling, Jim Kaput, Zohar Kerem, Nada Knežević, Paul Kroon, Rikard Landberg, Claudine Manach, Dragan Milenkovic, Ana Rodriguez-Mateos, Francisco A. Tomás-Barberán, Tom van de Wiele, Christine Morand

**Affiliations:** 1 The Rowett Institute, University of Aberdeen, Foresterhill, Aberdeen, AB25 2ZD UK; 2 VTT Technical Research Centre of Finland, PO Box 1000, Tietotie 2, Espoo, Finland; 3 Instituto de Biologia Experimental e Tecnológica, Apartado 12, Oeiras, Portugal; 4 Department of Nutrition and Preventive Medicine, Norwich Medical School, University of East Anglia, Norwich, UK; 5 Food and Health Laboratory. Research Group on Quality, Safety, and Bioactivity of Plant Foods, CEBAS-CSIC, Campus de Espinardo, Murcia, Spain; 6 UCD Institute of Food and Health, School of Agriculture and Food Science, University College Dublin, Dublin, Ireland; 7 Unilever Research and Development Vlaardingen, Vlaardingen, The Netherlands; 8 Vydiant, Sacramento, CA 95816 USA; 9 R.H. Smith Faculty of Agriculture, Food and Environment, The Hebrew University of Jerusalem, Jerusalem, Israel; 10 Podravka d.d, 48000 Koprivnica, Croatia; 11 Quadram Institute Bioscience, Norwich Research Park, Norwich, UK; 12 Division of Food and Nutrition Science, Department of Biology and Biological Engineering, Chalmers University of Technology, Gothenburg, Sweden; 13 INRA, UNH, Unité de Nutrition Humaine, CRNH Auvergne, Université Clermont Auvergne, Clermont-Ferrand, France; 14 Department of Nutritional Sciences, Faculty of Life Sciences and Medicine, School of Life Course Sciences, King’s College London, London, UK; 15 Faculty of Bioscience Engineering, Center for Microbial Ecology and Technology, Ghent University, Ghent, Belgium

**Keywords:** Healthy diet, Cardiometabolic diseases, Inter-individual variability in responses, Stakeholders, Food industry

## Abstract

**Background:**

A healthy diet and optimal lifestyle choices are amongst the most important actions for the prevention of cardiometabolic diseases. Despite this, it appears difficult to convince consumers to select more nutritious foods. Furthermore, the development and production of healthier foods do not always lead to economic profits for the agro-food sector. Most dietary recommendations for the general population represent a “one-size-fits-all approach” which does not necessarily ensure that everyone has adequate exposure to health-promoting constituents of foods. Indeed, we now know that individuals show a high variability in responses when exposed to specific nutrients, foods, or diets.

**Purpose:**

This review aims to highlight our current understanding of inter-individual variability in response to dietary bioactives, based on the integration of findings of the COST Action POSITIVe. We also evaluate opportunities for translation of scientific knowledge on inter-individual variability in response to dietary bioactives, once it becomes available, into practical applications for stakeholders, such as the agro-food industry. The potential impact from such applications will form an important impetus for the food industry to develop and market new high quality and healthy foods for specific groups of consumers in the future. This may contribute to a decrease in the burden of diet-related chronic diseases.

## Key messages

- Individual differences in ADME (Absorption, Digestion, Metabolism and Excretion) is believed to underpin much of the inter-individual variation in responses.
- Recent developments in the area of food metabolome databases and fast improvements in innovative metabotyping technologies hold great promise for improved profiling of dietary intake, exposure to individual ingredients, foods and dietary patterns, as well as our ability to identify individual responsiveness.
- The food industry needs well-defined population clusters or targets in order to be able to design “personalized products”.
- There are indeed excellent industrial opportunities for foods that modulate gut microbiota, and thereby enable the delivery of food bioactive metabolites.
- It is currently not clear whether knowledge on individual nutrient needs, based on genetic or metagenomic data, would affect long-term dietary and health behaviours.
- Data to support the development of dietary recommendations may need to be generated by new n-of-1-based study designs in the future.

## Inter-individual variability in cardiometabolic response to consumption of plant bioactives

The COST Action POSITIVe, a multidisciplinary and cross-sectorial European network of top-level scientists from more than 70 research institutions in 32 countries, members of regulatory authorities, and representatives of the food industry, has been instrumental in highlighting the large magnitude of individual variation in responses of health biomarkers to interventions with plant bioactives [1]. Variability in response to intervention is often not accounted for in studies, is considered an inconvenient impediment for establishing the efficacy of these plant bioactives in population-based studies, and is masked by common statistical methods that generate an average for the population rather than the individual results. Rarely are results reported for all individuals in a study to fully describe the extent of variation, but when they are, the extent of the variation can be seen to be dramatic. For example, total urinary excretion of naringenin phase-2 conjugates from a fixed dose of orange juice ranged from 1.6 to 59% (37-fold difference) in a study of 129 participants [2]. Individual differences in ADME (Absorption, Digestion, Metabolism, and Excretion), which includes the role of gut microbiota in the metabolism of plant polyphenols and other bioactives, is believed to underpin much of the inter-individual variation in responses.

For example, the clustering of urolithin metabotypes has helped to understand how inter-individual differences in the metabolism of pomegranate ellagitannins can be linked to individual differences in responsiveness in cardiovascular risk biomarkers [3]. In addition, a recent review that was executed as part of the COST Action POSITIVe highlighted the large range of factors, including age, BMI, sex, *Helicobacter pylori* infection, blood lipids, drug intake, microbiota as well as genetic polymorphisms (e.g., SNPs or single-nucleotide polymorphisms) that are known (and speculated) to influence carotenoid metabolism and exposure [4]. A different example shows that variability in the activity of polymorphic carriers and post-absorptive phase I and II metabolizing enzymes that depend on age, sex, or genotype are believed to contribute to the heterogeneity in flavonoid ADME. A significant part of the inter-individual variability in flavonoid ADME may be attributed to genetic variability and the gene–environment interactions, although it is currently unclear which gene variants and environmental factors contribute to responses [5]. Flavonoid ADME are also highly dependant on gut microbial metabolism—indeed, some of the microbiome-mediated bioconversions result in the production of metabolites with enhanced biological activity, with one of the best known examples being the conversion of soy isoflavones into equol [6]. Lignans constitute a similar example, where plant lignans are converted into mammalian lignans, enterdiol, and enterolactone by the gut microbiota. Main determinants of circulating enterolignans are plant lignan intake, composition, and activity of intestinal microflora, antimicrobial use, nutrient intake, BMI, smoking, sex, and age [7]. Polyphenols exemplify the complexity of metabolism of dietary secondary metabolites: computational analysis found that the most characterized 389 polyphenols interacted with 5699 unique proteins in an interactome of almost 12,000 interactions. These interactions mapped to a large number of diverse intermediary and regulatory pathways [8].

Inter-individual variation in response to food intake is a very recent development in nutrition and health. Knowledge of differences in metabolism of most plant food bioactives are currently limited to the inter-individual variances in plasma or urine concentrations of selected metabolites in controlled intervention studies. We only have scattered information on how the food matrix may affect the metabolism of plant food bioactives. In addition, molecular markers and genes of importance in ADME are typically unknown for individual compound groups. Extensive literature research completed as part of COST Action POSITIVe revealed a significant deficit in knowledge about the carriers, enzymes/isoforms, and gut bacteria involved in absorption and metabolic pathways, making it difficult to identify key molecular determinants of ADME variability. Furthermore, a limited number of clinical trials have suggested that metabolic or related disease traits of individuals can also influence the responsiveness to the consumption of plant sterols and some polyphenols [9].

The COST Action POSITIVe group identified a number of common factors from published human studies that appear to affect responsiveness to plant food bioactives. Several meta-analyses suggested that the magnitude of the responses was generally larger in those who were overweight and in subjects at risk of, or currently diagnosed with cardiovascular disease (CVD). For example, the consumption of foods and/or derived products containing flavanols has been associated with a significant decrease in body mass index (BMI), waist circumference, and total and low-density lipoprotein (LDL) cholesterol in individuals with a BMI ≥ 25.0 kg/m^2^ [10]. Similarly, in overweight and obese populations, the intake of food products containing ellagitannins was associated with a reduction in waist circumference, total and LDL cholesterol, triglycerides, glucose, and systolic blood pressure (SBP). Similarly, the intake of anthocyanin-containing products was associated with a significant reduction in both systolic (SBP) and diastolic blood pressure (DBP) [11]. These types of results indicate that overweight subjects may constitute a target group who would benefit most from the intake of these bioactive compounds.

Although numerous systematic reviews and meta-analysis have revealed that inter-individual variation in response to plant polyphenols is a real issue that warrants further investigation, the underlying concepts and methods of meta-analyses yield limited insights into determinants that drive responsiveness to dietary intervention in individuals. Indeed, many of the large randomised controlled trials (RTCs) have demonstrated that only ~ 40% of the trial participants respond to dietary interventions [12]. Currently, the nutrition and food research communities have not conducted the appropriately designed interventions, nor do they have the databases that would allow data fusion between studies or the grouping of individual data. Translating population attributable risks to individual risks or benefits has not been accomplished except for a few examples [13]. Despite this, a wide range of newly launched apps and tools create the impression that effective personalised diets can be recommended based on limited genetic and phenotypic profiles. These efforts are likely premature [14, 15] and could potentially damage the scientific reputation of the field and the ability for investments in the field of personalised and precision nutrition.

## What is needed to deliver future impact for stakeholders?

**Fig. 1:**
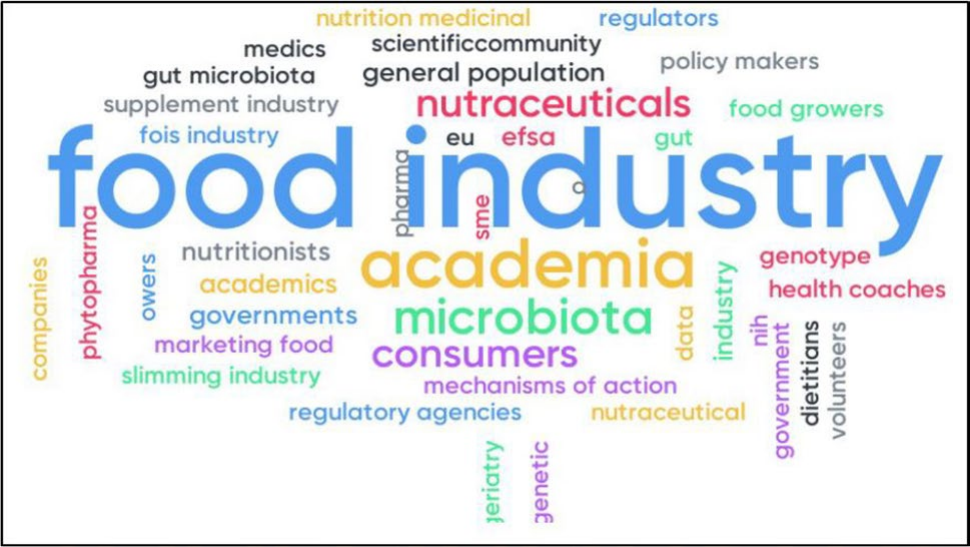
Digital Mentimeter Word Cloud revealing the most important stakeholders of the research field of inter-individual variability in response to diet

To identify the current opportunities and future needs for translation of scientific knowledge on inter-individual variability in response to dietary bioactives into practical applications for stakeholders, we established the main stakeholders of the research field using a digital Mentimeter Word Cloud during the final COST Action POSITIVe meeting in September 2019 (Fig. 1). The attendees consisted of a multidisciplinary and cross-sectorial European network of top-level scientists (nutritionists, clinicians, geneticists, epidemiologists, microbiologists, experts in gut microbiome and nutrigenomics, bioinformaticians, molecular biologists, and food scientists) from more than 70 research institutions in 32 European countries, members from regulatory agencies and representatives of the food industry. The results revealed that the food industry was perceived to be one of the most important stakeholders in this field, closely followed by academia, the nutraceutical industry, consumers, and to a lesser extend policy makers and regulatory agencies.

**Fig. 2:**
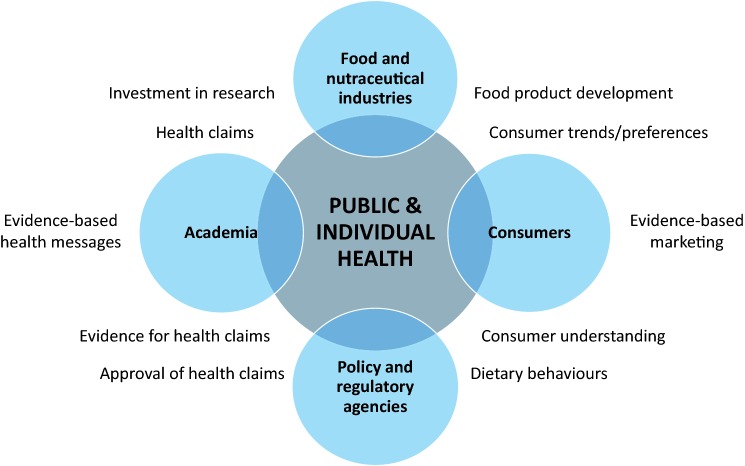
Schematic overview of main stakeholders and collective key impacts

Here, we will discuss available tools, perceived limitations and requirements for each of these stakeholders groups to create collective impact on individual and public health outcomes in the future (Fig. 2).

### Food and nutraceutical industry

The challenge for the food industry is to develop processed food products that are shelf-stable, inexpensive, attractive for consumers, and that contribute to healthier diets. Ideally, foods would be both desirable and healthy. However, consumers often select products that are not based on their needs to maintain or improve health. As such, it is important for the food industry to better understand consumer preferences, but also how these can be linked to scientifically proven health benefits. Developing, marketing, and selling these products would have the highest impact on individual and public health. However, regulations governing nutrition and health claims are mostly focused on health effects of individual nutrients in well-defined target groups. The result has been an overemphasis on single-functional ingredients as the basis for petitioning for health claims. Applications to regulatory agencies for health claims on foods and drinks throughout the European Union (Regulation 1924/2006 [16]) are expensive and time consuming, and require multiple rigorous scientific studies to substantiate a single claim. This is a disadvantage in a competitive market and, therefore, may discourage companies from making such investments. Nevertheless, the development of evidence-based marketing messages conveying positive health messages, advocating balanced intakes of manufactured and fresh foods that also account for nutrient security and sustainability, would contribute to short- and long-term needs of industry marketing strategies. Indeed, it is vitally important for a food industry that information from health claims can actually affect consumer understanding, purchase, and consumption [17]. The requirements for generating evidence for healthier foods limit research to larger companies that have sufficient revenue for long-term investment in research programmes. These often attempt to understand the entire chain from molecular mechanisms to evidence from human intervention studies. The timeline for such research intensive programmes is often too long to account for the rapidly changing consumer “preferences”. Examples are products that are gluten-free, organic, alternative protein or packaged in sustainable containers.

**Fig. 3:**
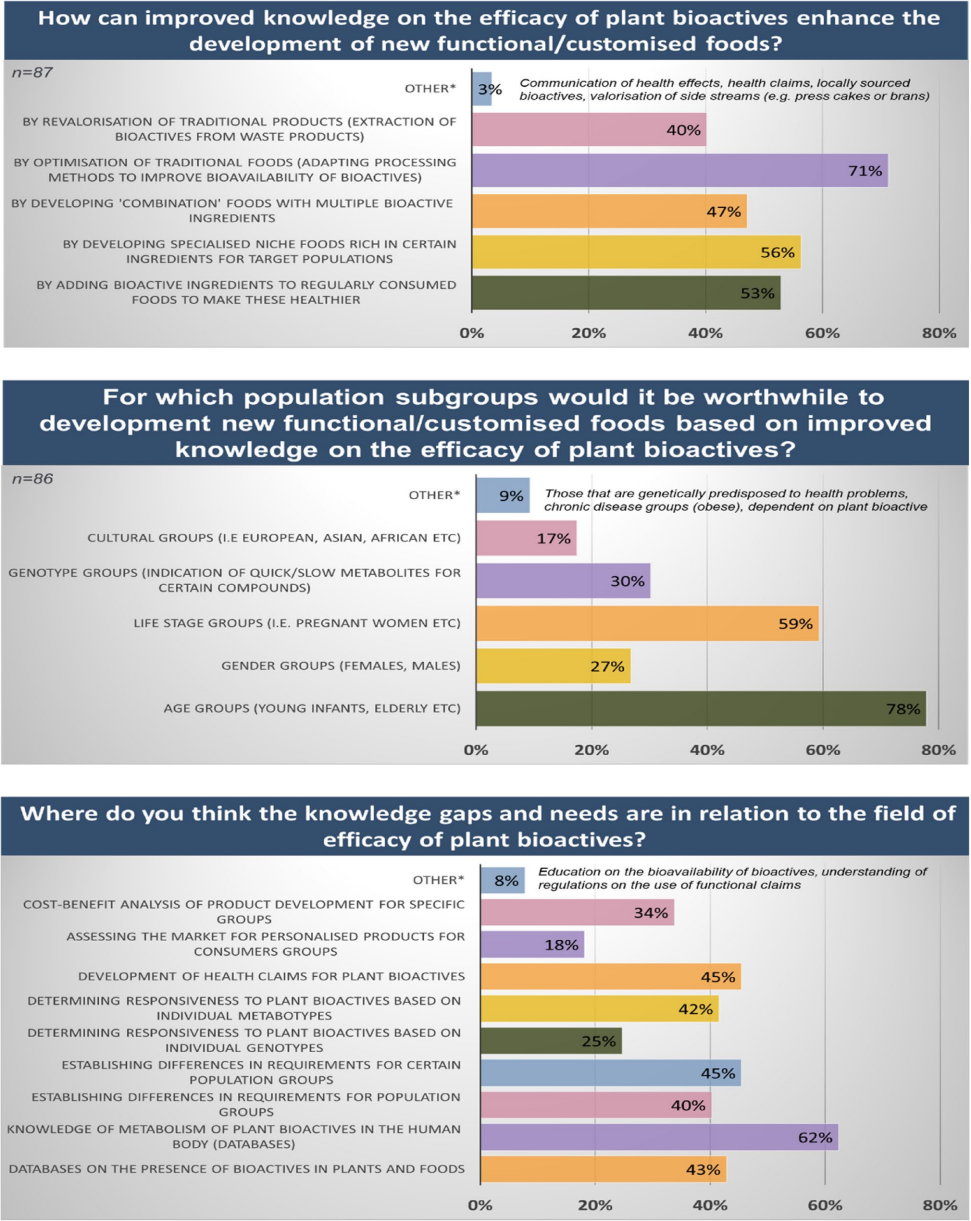
Survey outcomes highlighting key opportunities and requirements for the field of dietary plant bioactives amongst 84 key stakeholders from the food and drink sector, SMEs, dissemination organisations, public health bodies, food distributors, raw material suppliers, trade associations, and pharmaceutical companies

The challenges involved in the development of healthy foods, health claims, and marketing messages become even more complicated when considering a market, where the demand for personalised products has increased in recent years. In order for this new trend to prosper while supporting public health, the food industry needs to have well-defined population clusters or targets. These targets should extend beyond those defined by sex or age, and lead the design of “personalised products”. A survey across 84 stakeholders from large food companies (40%), the European food and drink sector (36%), SMEs (13%), dissemination organisations (6%), public health bodies (4%), and other stakeholders (food distributors, raw material suppliers, trade associations, pharmaceutical companies; 15%) highlighted that the majority of stakeholders and end-users believe that improved knowledge on the efficacy of plant bioactives can help to optimise product development. It is also shared by many that improving traditional foods—for example, by improving processing methods to increase bioavailability of health-promoting compounds from plants—will prove successful. The POSITIVe group also concludes that improved knowledge on the efficacy of plant bioactives can help to optimise product development, especially for specific age groups and lifestyle groups. This knowledge should support the extension of the range of popular products to provide personalised benefits to the targeted populations. One of the largest knowledge gaps in the research field is perceived to be knowledge of metabolism of plant bioactives in the human body, presented in databases (Fig. 3). There are indeed excellent industrial opportunities for foods that modulate gut microbiota, and thereby enable the delivery of food bioactive metabolites [18]. Moreover, the microbiota of the host appears to be an important determinant of the health effects of specific foods, and thus, assessment of subjects’ habitual microbiota or biomarkers thereof may be one strategy for tailoring foods for optimal effects across groups of individuals [19, 20].

### Academia

In general, we lack studies that provide data on both the bioavailability of, and biological responsiveness to, plant bioactives, especially after standardized intake of plant bioactives [21, 22]. Studies are usually too small to allow the identification of common factors influencing ADME, or to stratify subjects based on responsiveness to identify effects and mechanisms in more homogenous groups. Furthermore, many studies rely on food intake data which does not necessarily relate to individual exposure to, and bioavailability of, plant bioactives. Last but not least, we lack robust and database-driven opportunities for data fusion across RCTs that allow aggregation of individual data to identify factors that explain inter-individual variation in cardiometatobolic responses to plant bioactives.

The field would benefit from opportunities, where inter-individual variability can be investigated in cohort studies that facilitate multiple longitudinal samplings and metabolic profiling of blood, urine, and the gut microbiome to ascertain cardiometabolic responsiveness. In addition, the field would benefit from large RCTs that collect information on individual responsiveness to build the robust evidence-base to mechanistically understand health benefits and establish a cause and effect relationship. Study designs of such RCTs should allow for comprehensive baseline profiling and measure genetic variation, gut microbiota composition, lifestyle, and environment [23]. Recent developments in the area of metabolomics as well as food metabolome databases, and fast improvements in innovative metabotyping technologies [24–26] hold great promise for our ability to profile dietary intake and exposure to individual ingredients, foods and dietary patterns, as well as our ability to identify individual responsiveness [27]. Combining these data sets would improve the ability to establish high probability associations. Indeed, metabotyping has been proposed as a stratification tool based on ADME capacity, as has been done for equol and non-equol producers after intake of soy isoflavones, urolithin metabotypes after the intake of pomegranate ellagitannins [21], or for the urinary excretion of prenyl naringenin after hops flavanone intake [28].

Data fusion approaches exploiting studies that have been published, or are underway, may allow for the creation of larger data sets that would have sufficient statistical power to reveal relationships between endogenous factors and cardiometabolic health outcomes on the individual level. Such approaches would also allow the implementation of novel computational methods to extend additive genetic risk scores to include nonlinear gene–gene, gene–environment, and epigenetic interactions that influence responses to plant bioactives and other nutritional variables [29]. However, we found that data fusion approaches were inherently difficult because of ethical and logistical factors that limit access to data and that differences in study designs and outcomes make it almost impossible to merge data. Notwithstanding, such an approach is of great and common interest to researchers working in this area, since it facilitates subgroup analyses and gathers evidence for factors driving responsiveness in individuals or groups of subjects that share similar characteristics. Indeed, initiatives such as the European Joint Programming Initiative (JPI; https://ec.europa.eu/programmes/horizon2020/en/h2020-section/joint-programming-initiatives) a Healthy Diet for a Healthy Life require that data collected by funded consortia are made publicly available. Similarly, the medical research area has been working on the establishment of specific requirements for third-party access to anonymized individual data from clinical trial participants [30]. This is not a trivial issue, but similar mechanisms to achieve data integration in nutrition research should be pursued by the combined efforts of researchers, clinical trial units, nutritional journals, and international platforms. The sum of these considerations leads to a requirement for additional and extensive funding to perform new human studies which include the conceptual and methodological approaches to analyse variability in response.

### Consumers

Recent evidence suggests that a personalised approach based on an individual’s diet, phenotype, and environment improves healthy eating patterns and choices, at least in the short-term and in a research setting [31]. Such personalised advice may coincide with an individual’s specific needs and habits increasing the feeling of involvement and creating a higher awareness. The public appears to be interested in the adoption of personalised nutrition services, including affordable, simple, and reliable gadgets for self-classification and self-monitoring. Direct-to-consumer DNA testing for personalised diets has spurred extensive scientific and ethical debates in the literature about the underlying knowledge base for interpreting the results. In addition, consumers may have reservations about a service providers’ ability to ensure the secure handling of their personal health data [32]. A recent study revealed that health apps pose significant privacy risks—sharing of private medical user data is routine, yet far from transparent for users and clinicians [33]. A Europe-wide study showed that consumersʼ intention to adopt personalised nutrition services depends more on perceived benefit and effectiveness than on perceived privacy risk. However, services requiring information of an individual’s DNA-raised consumers’ perceived privacy risk without increasing perceived benefit [34]. Whether knowledge of an individual’s nutrient needs based on genetic or metagenomic data would affect long-term dietary and health behaviours is still unknown. The recent Food4Me study showed that the MTHFR genotype did not significantly improve intakes of dietary folate [35], and a meta-analysis revealed no significant effects of communicating DNA-based risk estimates on health behaviours such as smoking, diet, physical activity, alcohol and medication use, or indeed on motivation to change behaviour [36].

Most current personalised nutrition approaches provide dietary advice based on existing food products. Targeting new personalised foods to individuals or groups of individuals would have to be produced, marketed, and distributed to increasingly small consumer segments. The financial viability of products targeted at the group level is uncertain [37]. Indeed, personalised foods with controlled ingredient formulation will be more challenging to produce using existing manufacturing processes. Recent developments in emerging technologies (such as 3D printing) may provide new opportunities for the production of personalised foods with specific functional properties that meet personal requirements and expectations in relation to flavour, colour, shape, and texture [38]. This could make personalized nutrition more appealing to consumers and to the food industry in the future.

### Policy makers and regulatory agencies

Current processes of regulatory agencies are not consistent with personalisation of recommendations and dietary advice. Dietary guidance is currently based on classical endpoint studies using population-based statistics. On the other hand, personal and precision nutrition consider individual requirements based on individual consumer needs and preferences. Data to support the development of recommendations may be generated by new n-of-1-based study designs [39]. The development of new regulatory pathways beyond the reliance on RCTs with classical endpoints may be necessary, possibly including novel approaches assessing individuals’ responses that produce replicated results [13]. Developing machine-learning regulations will be necessary to ensure replicability of data analysis of research and real-world data. An interim step would be to focus on nutritional guidelines for different population groups defined by data consisting of social, lifestyle, and environmental factors.

Another level of complexity for personalisation of recommendations and dietary advice is that guidelines consider whole foods and dietary patterns rather than single nutrients or foods. Although it may seem intuitive that responses to a single nutrient may produce more replicable results, such approaches assume an inflexible metabolic machinery inconsistent with first-principles of biological systems that flexibly respond to changing environments. In addition, one nutrient rarely has a single main mechanism or effect. Omics technologies now permit the elucidation of how combinations of nutrients in foods produce specific metabolic readouts. Nevertheless, developing health claims of the beneficial effects of whole foods and/or diets is challenging and will require new research designs and regulatory guidelines for complex mixtures rather than individual nutrients.

## Conclusion

Progress in the field of personalised and precision nutrition aims for a better prediction of individual responses rather than population-based averages. Our current knowledge was based on statistical approaches and study designs that masked inter-individual variability in response to dietary bioactives. Here we make a strong case for more longitudinal and clinical intervention studies that account for inter-individual variability, with the scope of creation of databases that would allow data aggregation, stratification, and fusion to identify how common factors influence ADME and responsiveness to dietary plant bioactives. The knowledge coming from such studies and a collaborative vision to translate results of these efforts to individual and public health will facilitate the establishment of evidence based, and hence publicly acceptable, health claims that shall support effective marketing strategies to increase consumer understanding and consumption of healthier foods (Fig. 2).

## Abbreviations

ADME: Absorption, digestion, metabolism and excretion
BMI: Body mass index
COST: European cooperation in science and technology
CVD: Cardiovascular disease
JPI: Joint-programming initiative
LDL: Low-density lipoprotein
POSITIVe: Inter-individual variability in cardiometabolic response to consumption of plant bioactives
RCT: Randomised clinical trials
SNP: Single-nucleotide polymorphism
